# Evidence that 6q25.1 variant rs6931104 confers susceptibility to chronic myeloid leukemia through *RMND1* regulation

**DOI:** 10.1371/journal.pone.0218968

**Published:** 2019-06-25

**Authors:** Young Min Woo, Sehwa Kim, Jong-Ho Park, Nan Young Lee, Jong-Won Kim, Dennis Dong Hwan Kim

**Affiliations:** 1 Department of Health Sciences and Technology, Samsung Advanced Institute for Health Sciences and Technology, Sungkyunkwan University, Seoul, Korea; 2 Department of Laboratory Medicine, Kyungpook National University Chilgok Hospital, Daegu, Korea; 3 Samsung Advanced Institute for Health Sciences and Technology, Sungkyunkwan University, Samsung Medical Center, Seoul, Korea; 4 Department of Laboratory Medicine and Genetics, Samsung Medical Center, Sungkyunkwan University School of Medicine, Seoul, Korea; 5 Department of Medical Oncology & Hematology, Princess Margaret Cancer Centre, University Health Network, University of Toronto, Toronto, Canada; University of British Columbia, CANADA

## Abstract

Chronic myeloid leukemia (CML) is a clonal myeloproliferative disorder. Our previous study reported novel loci as genetic markers associated with increased susceptibility to CML. The present study conducted an expression quantitative trait loci (eQTL) analysis to confirm that the single nucleotide polymorphisms (SNPs) at these loci affect the expression of candidate CML-susceptible genes. We identified that three SNPs (rs963193, rs6931104, and rs9371517) were related to the gene expression pattern of *RMND1* (Required For Meiotic Nuclear Division 1 Homolog) in both granulocytes and mononuclear cells from 83 healthy donors. Furthermore, reduced expression of *RMND1* expression was noted in CML patients compared with that in healthy individuals. We used the eQTL browsing tool to assess the regulatory information on the three associated significant SNPs, out of which rs6931104 showed strong evidence of regulatory effects. Chromatin immunoprecipitation (ChIP) assays demonstrated that A alleles of rs6931104 could significantly change the binding affinity of transcription factor (TF) RFX3 compared to the G alleles. Then, we performed *in vitro* experiments on *BCR-ABL1*-positive (*BCR-ABL1*^+^) cell lines. We found that expression of the CML-susceptible gene *RMND1* is affected by the binding affinity of TF RFX3, suggesting that RFX3 plays a role in *RMND1* expression. Our findings suggest potential target genes for associations of genetic susceptibility risk loci and provide further insights into the pathogenesis and mechanism of CML.

## Introduction

Chronic myeloid leukemia (CML) is a clonal disorder in hematopoietic stem cells characterized by the presence of an oncogenic *BCR-ABL1* fusion gene that encodes a constitutively activated fusion protein carrying tyrosine kinase activity. To date, genome-wide association studies (GWASs) have revealed that single nucleotide polymorphisms (SNPs) are associated with CML development, progression, and treatment outcome [1–4]. However, beyond the established statistical associations, the functional relevance or underlying mechanisms have rarely been explored.

In our previous study, we performed a genome-wide analysis and reported novel loci associated with susceptibility to CML. In particular, the SNPs at the 6q25.1 locus were associated with CML in cohorts of both Korean and European descent, while the SNPs at the 17p11.1 locus were associated with CML in the Korean cohort only. In the present study, to understand the functional role of these SNPs, we conducted expression quantitative trait loci (eQTL) and functional analysis of related transcription factor in confirming whether the SNPs in these loci affect the expression of CML susceptibility candidate genes.

## Materials and methods

### Study design and description of participants

**Fig 1:**
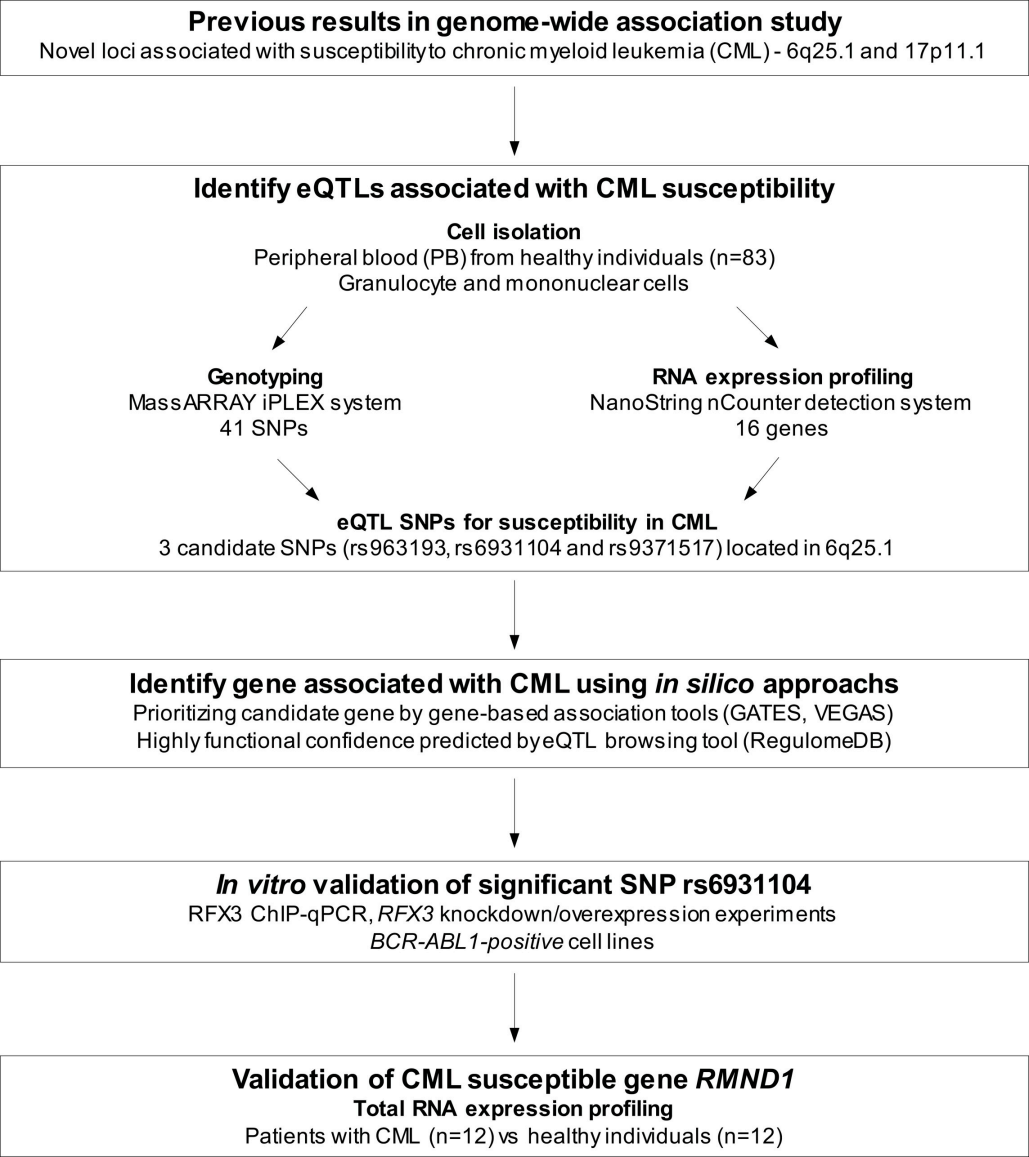
Overall study design and workflow. To identify the eQTLs associated with CML susceptibility, we performed genotyping, RNA expression profiling, *in silico* analysis, and functional validation using healthy individuals, CML patients, and *BCR-ABL1*-positive human cell line models.

The study was approved by the Institutional Research Board of the Samsung Medical Center, Seoul, Korea. In the initial experiments, we have applied an eQTL approach in both granulocytes and mononuclear cell fraction separately to evaluate different gene expression according to the cell subsets. A total of 83 healthy donors (24 male and 59 female) who do not have any previous history of blood disorders and normal blood counts were consented and collected from March to September 2012 for this study. Granulocytes and mononuclear cells collected from peripheral blood (PB) were isolated using the Ficoll density gradient method. DNA and RNA were extracted within 24 hours after venipuncture. In order to compare the expression level of a candidate gene with healthy individuals, we additionally recruited 12 patients with CML on imatinib treatment consented for future research from March to April 2013 (Fig 1).

### Genotyping

Before evaluating whether the SNPs identified in the GWAS were functional with respect to gene expression level, we determined the genotypic variation in whole blood samples from 83 healthy individuals. Yields of pure, double-stranded genomic DNA were obtained using the QIAamp DNA Blood Maxi Kit (Qiagen, California, USA). All steps were followed according to the manufacturer’s instructions. The genomic DNA of the 83 samples was quantified by a NanoDrop ND-1000 Spectrophotometer (Thermo Fisher Scientific, Massachusetts, USA) and diluted to 10 ng/μl. We selected 41 SNPs with *p*-values less than 0.005 selected from the discovery set in our previous study for genotyping in two loci 6q25.1 and 17p11.1, respectively [5], and added 2 SNPs (rs3736175 and rs721348) known as expression quantitative nucleotides (eQTNs, http://eqtl.uchicago.edu/cgi-bin/gbrowse/eqtl/), all from these regions (Table 1). Genotyping of the 41 SNPs was performed on a MassARRAY iPLEX system (Agena Bioscience, California, USA). The MassARRAY Assay Design 3.1 software was used to design multiplex primers for each SNP.

**Table 1:** 41 SNPs selected for genotyping.

Chromosome	rs ID	Position^a^	*p*-value^b^
**6q25.1**	rs963193	151789355	4.32E-06
**6q25.1**	rs6931104	151786177	5.53E-06
**6q25.1**	rs9371517	151739810	4.05E-05
**6q25.1**	rs4869742	151907748	0.000415
**6q25.1**	rs3900024	151706570	0.0004525
**6q25.1**	rs7765741	151717302	0.0008366
**6q25.1**	rs6557140	151758042	0.002534
**6q25.1**	rs2295041	152773194	0.00293
**6q25.1**	rs9371600	152790533	0.003167
**6q25.1**	rs4391273	151893073	0.004039
**6q25.1**	rs9397105	152772494	0.00412
**6q25.1**	rs9397510	152797065	0.004507
**6q25.1**	rs7747960	152791474	0.004618
**6q25.1**	rs7751588	152797353	0.004929
**17p11.1**	rs2061893	25396146	6.47E-06
**17p11.1**	rs12950376	25541572	6.47E-06
**17p11.1**	rs1824900	25472214	6.82E-06
**17p11.1**	rs11080053	25388276	6.92E-06
**17p11.1**	rs10163524	22108783	7.09E-06
**17p11.1**	rs1828999	22217883	8.02E-06
**17p11.1**	rs7221571	25363654	8.18E-06
**17p11.1**	rs16972661	25398037	8.21E-06
**17p11.1**	rs1975980	25395691	9.70E-06
**17p11.1**	rs7213631	22120732	1.21E-05
**17p11.1**	rs1824901	25472565	1.25E-05
**17p11.1**	rs33962847	22084950	1.27E-05
**17p11.1**	rs1402724	25409526	1.37E-05
**17p11.1**	rs2313050	25346692	1.79E-05
**17p11.1**	rs11080056	25404049	2.42E-05
**17p11.1**	rs9972902	25455185	2.42E-05
**17p11.1**	rs7210668	22168795	2.51E-05
**17p11.1**	rs4795519	25541278	2.87E-05
**17p11.1**	rs2013347	22171190	3.44E-05
**17p11.1**	rs2340408	22235650	3.50E-05
**17p11.1**	rs2169956	25470744	3.78E-05
**17p11.1**	rs1520020	22105110	4.35E-05
**17p11.1**	rs12946383	21984361	0.0007233
**17p11.1**	rs8072903	21991614	0.0008935
**17p11.1**	rs7406339	22002556	0.001123
**Chromosome**	**rs ID**	**Position**^**a**^	**Related gene**
**6q25.1**	rs3736175	151773164	*RMND1*
**17p11.1**	rs7213148	25639046	*WSB1*

39 SNPs associated with CML susceptibility [5] and 2 SNPs (rs3736175 and rs721348) known as expression quantitative nucleotides (eQTNs) from these regions were selected.

^a^Physical position based on the human reference genome build hg19 (GRCh37).

^b^*p*-value of the discovery set in the previous study [5]

### Gene-based association analysis

We applied a gene-based association approach to prioritize susceptible genes in 6p25.1 locus associated with CML as combining the single SNP-based *p*-values using the discovery set (201 CML patients and 397 healthy individuals). We also validated the results of the gene-based approach in a validation set (356 CML patients and 1,000 healthy individuals) with Korean ethnicity.

The gene-based association test using extended Simes procedure (GATES) [6] is a rapid and powerful gene-based test to evaluate a gene with one or a few independent causal variants. We used the hg19 gene version to assign imputed SNPs. We built an analysis genome by rsID to optimize the annotations in the GATES dataset. The GATES tool considers that the pairwise SNP *p*-value correlation coefficient ρ is expected to be mainly determined by the pairwise linkage disequilibrium (LD) between the two corresponding SNPs as measured by the allelic correlation coefficient. We used HapMap JPT (Japanese) LD information data to calculate the correlation coefficient of the two SNPs. The SNPs were assigned to an extended gene region that was ± 50 kilobases (kb) from the 5′ and 3′ untranslated region (UTR).

The versatile gene-based association study (VEGAS) [7] method, which uses simulations from the multivariate normal distribution based on the LD structure, is a gene-level association test using the results from single SNP-based approach data [8]. We used the VEGAS software to test the 17,542 assigned genes with imputed SNPs by using the initial CML GWAS result data. To assign the SNPs to the genes, we defined the gene boundary within ± 50 kb from the 5′ and 3′ UTR, the same condition as for GATES. To consider the LD structure, we used HapMap JPT+CHB (Han Chinese) LD information data.

### Replication study for the gene-based approach at the 6q25.1 locus

The candidate genes identified at the 6q25.1 locus via gene-based approaches were tested in a new independent case-control replication set. To identify significant candidate genes, we selected those genes (i.e., *C6orf211*, *RMND1*, and *ZBTB2*) which had *p* < 0.001 in the discovery set. To validate the association with CML in the replication set, we selected five observed SNPs (rs6931104, rs9371517, rs3900024, rs7765741, and rs6557140) that had *p* < 0.005 at the 6q25.1 locus of the discovery set. The five SNPs were genotyped by the MassARRAY iPLEX system (Agena Bioscience) in the validation set (n = 1,356).

### mRNA expression profiling

For mRNA expression profiling, total RNA was extracted from each sample of granulocyte and mononuclear cell fractions using the TRIzol reagent (Invitrogen, California, USA). A total of 16 genes were tested including 9 genes located on the 6q25.1 and 17p11.1 loci (6q25.1: *MTHFD1L*, *AKAP12*, *ZBTB2*, *RMND1*, *C6orf211*, *C6orf97*, *ESR1*; 17p11.1: *FAM27L*, *WSB1*), as well as additional 7 genes (6q25.1: *IYD*, *PLEKHG1*, *SYNE1*; 17p11.1: *KCNJ12*, *KSR1*, *LGALS9*, *NOS2A*) located in the expanded region of these loci up to 500 kb from the target genes. Also, three internal reference genes, *GAPDH*, *OAZ1*, and *PPIA*, were added. Their mRNA expression levels were quantified using the NanoString nCounter amplification-free detection system [9]. All steps were followed according to the manufacturer’s instructions. Normalization of the mRNA expression was conducted by the nSolver software provided by NanoString Technologies.

### Functional prediction for eQTL SNPs

We used the RegulomeDB (http://regulome.stanford.edu) eQTL browsing tool to assess the regulatory information regarding the associated three significant eQTL SNPs at 6q25.1. The RegulomeDB score, which ranges from 1 to 6, is set up to assess the degree of confidence supporting a functional variant as a transcription factor (TF) binding site. A lower score indicates stronger evidence that an SNP affects binding and gene expression [10].

### Chromatin immunoprecipitation

We performed chromatin immunoprecipitation (ChIP) in 18 healthy individuals who did not have any previous history of blood disorder with normal blood counts. ChIP assays were performed using a Pierce Agarose ChIP kit (Thermo Fisher Scientific, Illinois, USA), and chromatin was cross-linked, fragmented, immunoprecipitated, and purified according to the manufacturer instructions using a rabbit polyclonal antibody against RFX3 (T-17, sc-10662; Santa Cruz Biotechnology, California, USA) or an anti-RNA polymerase II antibody (positive)/normal rabbit IgG (negative) as control (Thermo Fisher Scientific, Illinois, USA). Quantitative reverse transcription polymerase chain reaction (RT-qPCR; Applied Biosystems 7900 HT Real-Time PCR) was used to determine the ChIP assay results.

### Cell culture for *in vitro* assay

We used *BCR-ABL1*^+^ cell lines, including K562 (CCL-243; obtained from the American Type Culture Collection), CML-T1, and LAMA84 (ACC-7 and ACC-168; obtained from Deutsche Sammlung von Mikroorganismen und Zellkulturen GmbH). All cell lines were cultured in RPMI-1640 media (Invitrogen, California, USA) supplemented with 10% fetal bovine serum (FBS; Invitrogen, California, USA), 100 units/mL penicillin, and 100 μg/mL streptomycin (Invitrogen, California, USA).

### Gene knockdown and overexpression experiments

To further explore the link between *RMND1* gene expression and TF RFX3 binding affinity, we performed *RFX3* gene knockdown and overexpression experiments. K562, CML-T1, and LAMA84 *BCR-ABL1*^+^ cells were transfected with *RFX3* siRNA (L-011764-00-0005; ON-TARGETplus, SMARTpool, Dharmacon, Colorado, USA) and RFX3 overexpression vector (RC201137; True ORF Gold cDNA Clones, Origene, Maryland, USA). Non-silencing siRNA was also used as a negative control (51-01-14-04; Universal Negative Control DsiRNA, IDT, Iowa, USA). Each was transfected into the cells by electroporation using the Invitrogen Neon Transfection System as previously described [11]. The cells were then resuspended in medium containing 20% FBS and further incubated at 37°C. We performed RT-qPCR to evaluate *RFX3* gene knockdown or overexpression one day after transfection. We performed more than three independent experiments with at least triplicates to ensure accuracy.

### Gene expression analysis in CML patients and healthy individuals

To investigate the relationship between *RMND1* expression and CML, the *RMND1* mRNA level was assessed by RT-qPCR in CML patients and healthy individuals. Total cDNA was amplified using the SYBR Select Master Mix (Thermo Fisher Scientific, Texas, USA). After 45 cycles at 95°C for 5 sec and 60°C for 1 min, the dissociation curve analysis for ΔCt was analyzed using the SDS 2.4 and RQ Manager software provided by Life Technologies. For quantification, the RNA transcript expression was normalized by determining the ratio between the expression levels of the *RMND1* and *GAPDH* genes.

### Statistical analysis

We carried out an imputation analysis to fill in the missing genotype data using IMPUTE2 Version 2.3.0 [12, 13]. As a reference panel, the JPT + CHB (Phase II HapMap release #22 NCBI build 36) populations were used [14]. We also used the pre-phasing method SHAPEIT2 version v2.r727 for efficient imputation with a small loss in accuracy [8, 15]. The LD structure was assessed using Haploview version 4.2.7 [16]. The eQTL analysis was carried out using the ANOVA model provided by the Matrix eQTL software [17]. We applied the family-wise error (or *p*-value) correction using the False Discovery Rate (FDR) method. All statistical analyses were performed using R (R Foundation for Statistical Computing, Austria).

## Results

### *RMND1* expression is correlated with three SNP genotypes at 6q25.1

**Fig 2:**
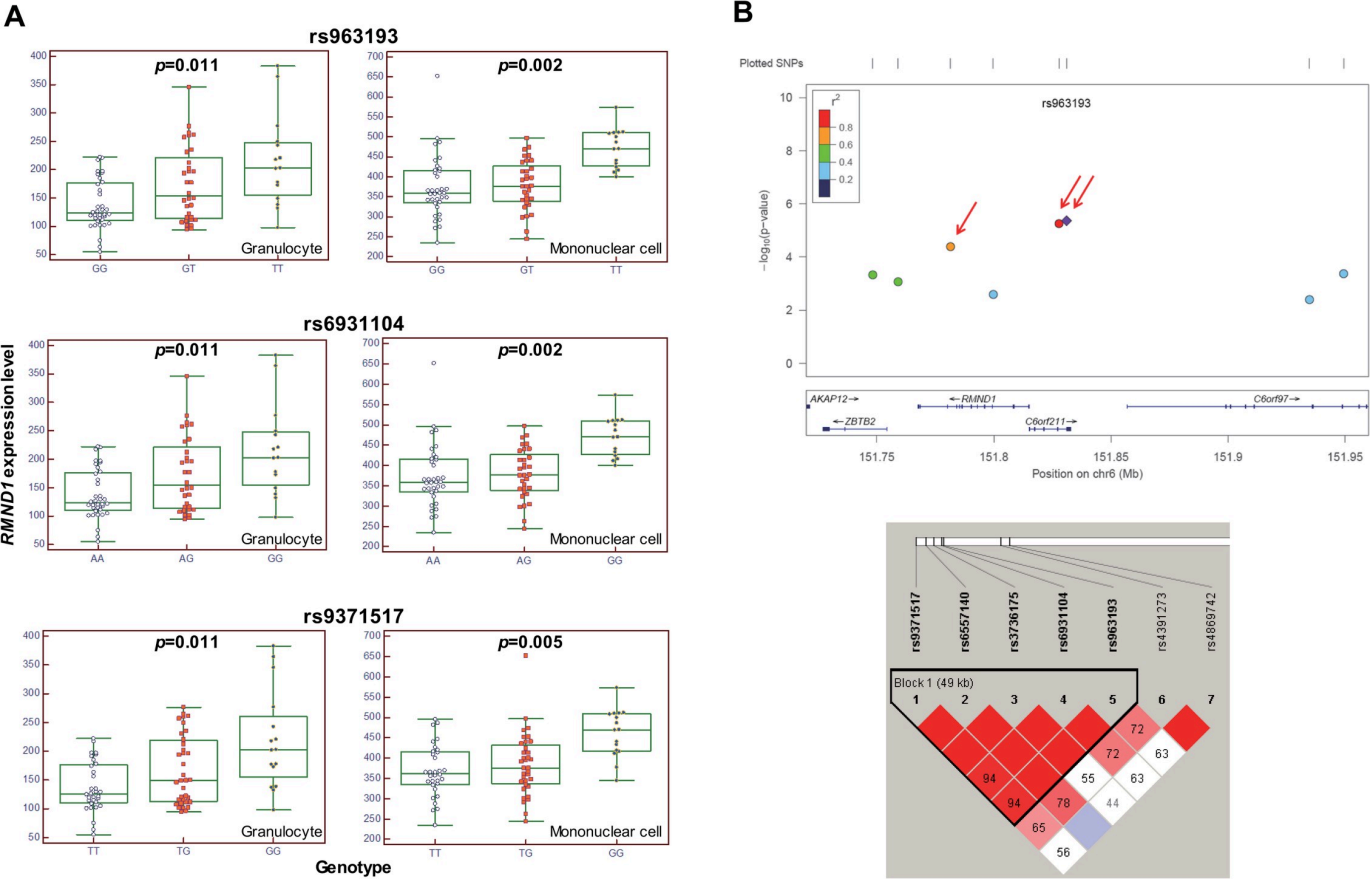
*RMND1* expression is correlated with the genotype of the functional SNP rs6931104 at 6q25.1. (A) Box-plot of the significant eQTLs in both cells. *RMND1* expression levels in granulocytes (left) and mononuclear cells (right) (corrected *p* < 0.05). (B) Manhattan plot and LD block of 6q25.1, including three significant SNPs (rs9371517, rs6931104, and rs963193). The Manhattan plot shows the *p*-value of the discovery set in the previous study, and the red arrows indicate these three SNPs. The LD block image was created based on the genotype information in this study.

We examined the associations of 41 SNPs at two loci, 6q25.1 and 17p11.1 (Table 1), in 83 healthy individuals to evaluate whether those SNPs were functional. We focused on nine genes that had previously been reported [5] in the regions of 6q25.1 (*MTHFD1L*, *AKAP12*, *ZBTB2*, *RMND1*, *C6orf211*, *C6orf97*, *ESR1*) and 17p11.1 (*FAM27L*, *WSB1*). Additional seven genes located up to 500 kb from the target genes were added (6q25.1: *IYD*, *PLEKHG1*, *SYNE1*; 17p11.1: *KCNJ12*, *KSR1*, *LGALS9*, *NOS2A*) to investigate any possible regulatory functions on the expression of neighboring genes. We found evidence that 3 candidate SNPs (rs963193, rs6931104, and rs9371517) located in non-coding regions of 6q25.1 were related to the gene expression pattern of *RMND1* in both granulocytes (corrected *p*: 0.011, 0.011, and 0.011, respectively) and mononuclear cells (corrected *p*: 0.002, 0.002, and 0.005, respectively). The genotype-dependent mRNA expression levels of *RMND1* showed similar patterns for rs963193 and rs6931104 (Fig 2A). A subset of significant SNPs identified as eQTLs were in strong LD, with an r^2^ value = 1. The LD block shows that the three SNPs are in the same block (Fig 2B). However, one SNP allocated to 17p11.1 was not found to be associated with the expression level of candidate genes at this locus.

### *RMND1* is significantly associated with CML in the two gene-based association tools, VEGAS and GATES

Using VEGAS, we identified three top-ranking genes (*RMND1*, *p* = 0.000011; *C6orf211*, *p* = 0.000016; and *ZBTB2*, *p* = 0.00029) in the candidate locus (6q25.1) associated with CML. We also confirmed the three genes associated with CML by another gene-based method, GATES, at the threshold *p*-value less than 0.001. Notably, *RMND1* (*p* = 0.011 for VEGAS and *p* = 0.0088 for GATES) and *ZBTB2* (*p* = 0.0093 for VEGAS and *p* = 0. 042 for GATES) were validated in an independent cohort of Korean subjects (total n = 1,356; case n = 356 and control n = 1,000) with more than one SNP with *p* < 0.005, which constituted original genotype, rather than imputed data. It is important to improve the probability that specific genes are on loci associated with CML because functional studies are not feasible for all located genes. In conclusion, our gene-based approach has identified the susceptible genes associated with CML.

### Non-coding variant rs6931104 has the strongest evidence of regulatory potential

We further investigated the regulatory effects (i.e., transcription factor binding sites, chromatin peak regions, and DNase accessibility) of the three eQTL SNPs (rs963193, rs6931104, and rs9371517) using RegulomeDB. Remarkably, rs6931104 (score = 1) is in LD with the significant *p*-value eQTLs rs963193 and rs9371517, SNPs that by themselves are not functional according to RegulomeDB (score = 6). rs6931104, the SNP with strong evidence of a regulatory function, is an intergenic SNP located in the *C6orf211* region and 12.9 kb upstream from the transcription start site (TSS) of *RMND1*. Specifically, RegulomeDB annotated rs6931104 as a putative binding site for the transcription factor (TF) of RFX3 (Regulatory Factor X3) (S1 Fig). Therefore, rs6931104 may be involved in the regulation of *RMND1* mRNA expression by regulating TF RFX3 binding.

### Change in RFX3 binding affinity is correlated with change in *RMND1* expression

**Fig 3:**
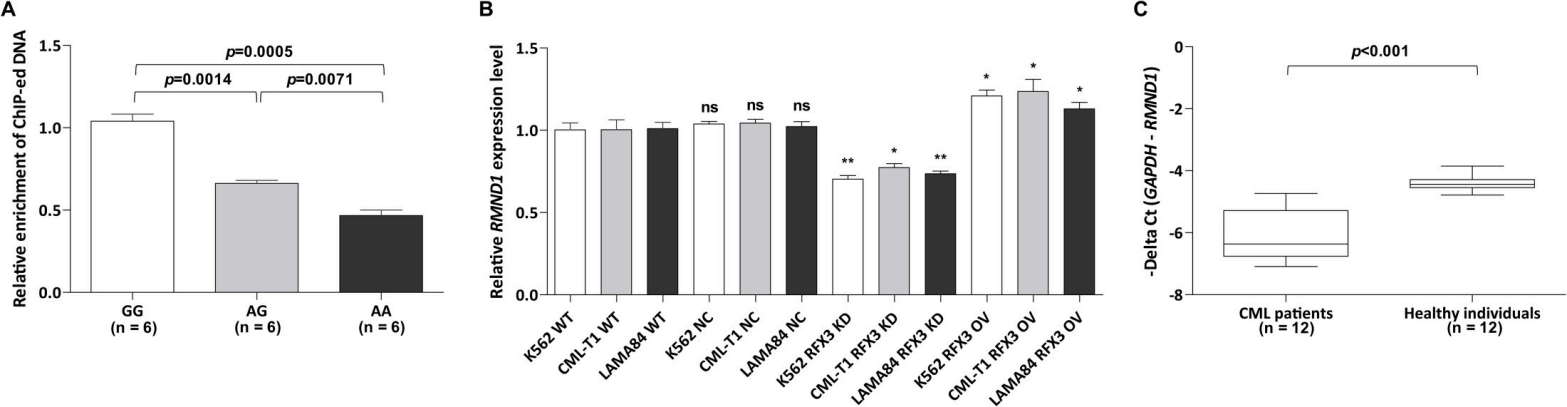
Expression of CML-susceptible gene *RMND1* is affected by TF RFX3 binding affinity. (A) The CML-associated SNP rs6931104 affects the binding affinity of transcription factor RFX3. ChIP-ed DNA enrichment determined by qRT-PCR is graphed as the amount of immunoprecipitated DNA. Error bars indicate SD values. (B) Effects of *RFX3* knockdown (KD) or overexpression (OV) on *RMND1* transcript levels in *BCR-ABL1*^+^ cells. Total RNA expression was assayed by RT-qPCR. Asterisk marked *p*-values were calculated by comparing with control in each of the three cell lines, respectively. **p < 0.01, *p < 0.05, ns–not significant. Error bars indicate SD values. (C) *RMND1* transcript levels in CML patients. Total RNA expression was assayed by RT-qPCR. Minus delta Ct (*GAPDH* Ct value–*RMND1* Ct value) indicates the expression levels (Y-axis) in samples from 12 human CML patients and 12 healthy individuals. All statistical significance was evaluated by the Student’s *t*-test.

To confirm this hypothesis, we performed ChIP-qPCR on 18 healthy individuals. As expected, the functional assays demonstrated that the presence of the A allele of the functional SNP rs6931104 showed the decreased binding affinity of the RFX3 transcription factor compared to the presence of the G allele. The amount of immunoprecipitated DNA was lower for either the AG or AA genotype compared to that for the GG genotype (Fig 3A). Thus, we postulated that the *RMND1* gene expression is dependent on the binding affinity of TF RFX3.

### RFX3 abundance is reflected in a change in *RMND1* expression in cell line models

To assess whether there was a correlation between *RMND1* gene expression and TF RFX3 abundance, we performed RFX3 gene knockdown and overexpression in *BCR-ABL1*^+^ cell lines. Using quantitative RT-PCR, *RFX3* knockdown was found to reduce *RMND1* mRNA levels compared to the respective control significantly. In contrast, *RFX3* overexpression resulted in a significant increase in *RMND1* mRNA abundance (Fig 3B). Using quantitative RT-PCR in the *BCR-ABL1*^+^ cells (K562, CML-T1, and LAMA84), we verified the decreased expression of *RMND1* with approximately 30%, 23%, and 27% compared to control, respectively. In *RFX3*-overexpressed cells, the quantitative RT-PCR results verified that *RMND1* expression levels were significantly increased by approximately 24%, 27%, and 16% in the stable *RFX3*-overexpressed cells compared to the control cells, respectively. These data suggest that transcriptional regulation by RFX3 is likely to affect *RMND1* expression.

### *RMND1* is downregulated in CML patients

*RMND1* expression levels were determined by RT-qPCR in samples from 12 human CML patients and 12 healthy individuals. As shown in Fig 3C, *RMND1* expression was significantly downregulated in CML patients compared to that in healthy individuals (*p* < 0.001). This finding on the relationship between the low *RMND1* levels and the CML samples supports the tumor-related role of *RMND1* in CML.

## Discussion

The eQTL study has emerged as an essential concept to investigate the biological effects of non-coding regulatory variants [18]. According to the results of the genotype-tissue expression (GTEx) project, nearly 50% of the genetic variants associated with human disease colocalize with an eQTL [19]. This means that eQTL analysis provides insights enabling a pathological understanding of non-coding variants associated with the disease.

The key finding of the present study is that the causal eQTL variant rs6931104 at 6q25.1 is capable of driving a differential expression of *RMND1* and that this action is at least in part dependent on the binding of TF RFX3. Several genes are located within a 1 Mb region of the 6q25.1 locus, including *ZBTB2*, *RMND1* [*C6orf96*], *C6orf97*, *C6orf211*, and *AKAP12* [5]. Recently, genome-wide studies have identified relationships in the expression patterns between genes and the SNPs in this region, which is upstream of the gene encoding ER (*ESR1*), which is associated with breast cancer susceptibility [20–24]. Dunbier and colleagues have previously suggested that some of the biological effects caused by *ESR1* could be mediated and modified by these co-expressed genes [21].

The CML-susceptible gene *RMND1* encodes a protein composed of 449 amino acids that localize to the mitochondria and is shown to behave as an integral membrane protein [25]. It belongs to the evolutionarily conserved proteins that share the DUF155 domain (Domain of Unknown Function 155) [26, 27]. However, its exact function remains unknown. Several studies have suggested that the *RMND1* gene is associated with human diseases, with lower *RMND1* expression correlating with breast cancer risk and worse relapse-free survival [28, 29], infantile encephalopathy, mitochondrial translation defects, hearing impairment, and renal failure [26, 30]. Moreover, *RMND1* orthologs are involved in cell division [31], and mitochondrial ribosomal proteins affect cell-cycle regulation [25, 32], suggesting a possibility that *RMND1* gene expression is associated with the proliferation of mature granulocytes and their precursors, a major characteristic of CML.

Non-coding SNPs that show associations with gene expression levels can constitute strong clues for the cause of certain diseases, such as SNPs located in transcription factor binding sites (TFBS) that can affect gene regulation by altering the binding affinity of the respective TFs [33, 34]. We demonstrated that the risk-associated SNP rs6931104 could significantly change the binding affinity of transcription factor RFX3. RFX3, also known as Regulatory Factor X3, has been characterized as a transcriptional activator that can bind DNA with other RFX family members [35, 36]. The biological relevance of RFX3 in disease has never been extensively investigated, and it is not known whether RFX3 is involved in tumorigenesis, particularly in CML. Thus, further functional studies of RFX3 are needed to elucidate the role of RFX3 in CML leukemogenesis based on the results of the current study.

In conclusion, we conducted an eQTL analysis and functional validation for lead variants and nearby genes using data from a genome-wide analysis on a large number of CML patients. The risk SNP rs6931104 lowers the binding affinity of TF RFX3; it also reduces the expression of *RMND1*. These results suggest that a reduction in the expression levels of *RMND1*, which serves as a tumor suppressor gene, is associated with CML pathogenesis. Theoretically, *RMND1* regulation can be a potential therapeutic strategy to improve treatment outcome in CML patients. In the present study, we focused on whether SNPs at a risk locus affected candidate gene expression levels using transcriptional eQTL. However, our results imply that other regulatory mechanisms may also be involved in the pathogenesis of CML. They also suggest that various types of modulation (i.e., chromatin modification QTL [37], chromatin interaction QTL [38], and translational QTL [39]) may affect CML pathogenesis. Further functional studies are needed to elucidate the role of other genes in CML leukemogenesis based on the results of the current study.

In our study, we have discovered additional biological mechanisms for understanding genetic susceptibility risk loci, and have provided a more profound insight into the genetic and biological basis for the pathogenesis of CML.

## Supporting information

S1 Fig rs6931104 showed strong evidence of regulatory effects. Functional information of rs6931104 (http://regulome.stanford.edu). SNPs categorized as '1f' were described to function as eQTLs and be related with either TF binding or DNase, which means that they have high functional confidence.
(PDF)

S2 Fig Effects of *RFX3* knockdown (KD) or overexpression (OV) on *RFX3* transcript levels in *BCR-ABL1*^+^ cells. Relative transcript levels of *RFX3* were quantified by RT-qPCR and normalized with a house-keeping gene *GAPDH*. The results show are representative of at least three independent experiments. Statistical analysis was performed using Student’s *t*-test. ***p < 0.001, ns–not significant. Error bars indicate SD values.
(PDF)

S1 Table Primer sequences used for *in vitro* assay. (XLSX)
