# Supplementary material for: Evidence that 6q25.1 variant rs6931104 confers susceptibility to chronic myeloid leukemia through RMND1 regulation
Source: PLoS One. 2019 Jun 25;14(6):e0218968. doi: 10.1371/journal.pone.0218968 (PMC6592567; doi:10.1371/journal.pone.0218968)
Supplement: S1 Fig — Functional information of rs6931104 (http://regulome.stanford.edu). SNPs categorized as '1f' were described to function as eQTLs and be related with either TF binding or DNase, which means that they have high functional confidence. (PDF) [file pone.0218968.s001.pdf]

Data supporting chr6:151786176 (rs6931104)

Score: 1f

Likely to affect binding and linked to expression of a gene target

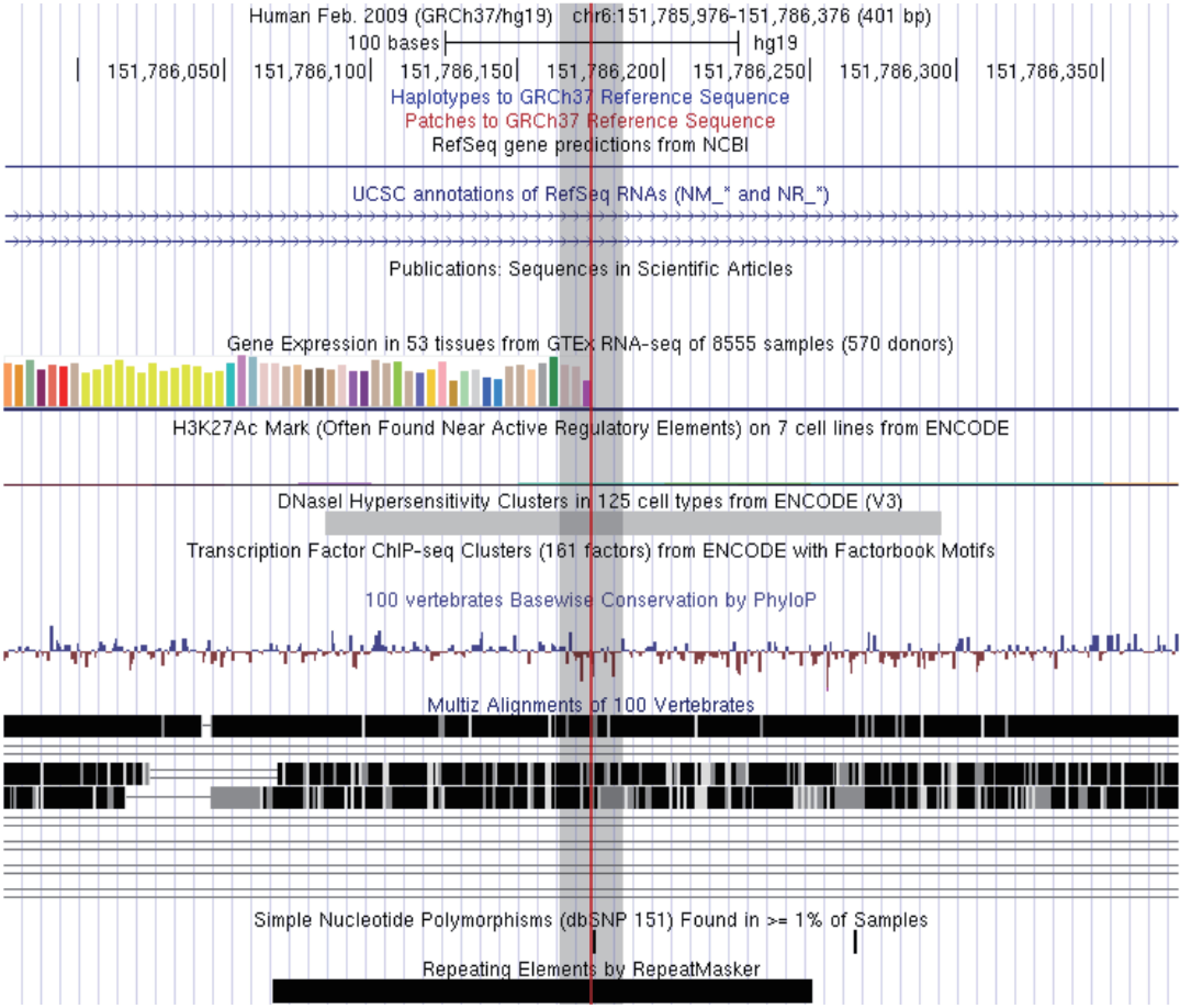

Protein Binding Filter:

Method	Location	Bound Protein	Cell Type	Additional Info	Reference
ChIP-seq	chr6:151786015..151786954	RFX3	K562		20378718

Single nucleotides Filter:

Method	Location	Affected Gene	Cell Type	Additional Info	Reference
eQTL	chr6:151786176..151786177	RMND1	Lymphoblastoid	cis	20220756
