# Supplementary figures and images for: Evidence that 6q25.1 variant rs6931104 confers susceptibility to chronic myeloid leukemia through RMND1 regulation
Source: PLoS One. 2019 Jun 25;14(6):e0218968. doi: 10.1371/journal.pone.0218968 (PMC6592567; doi:10.1371/journal.pone.0218968)

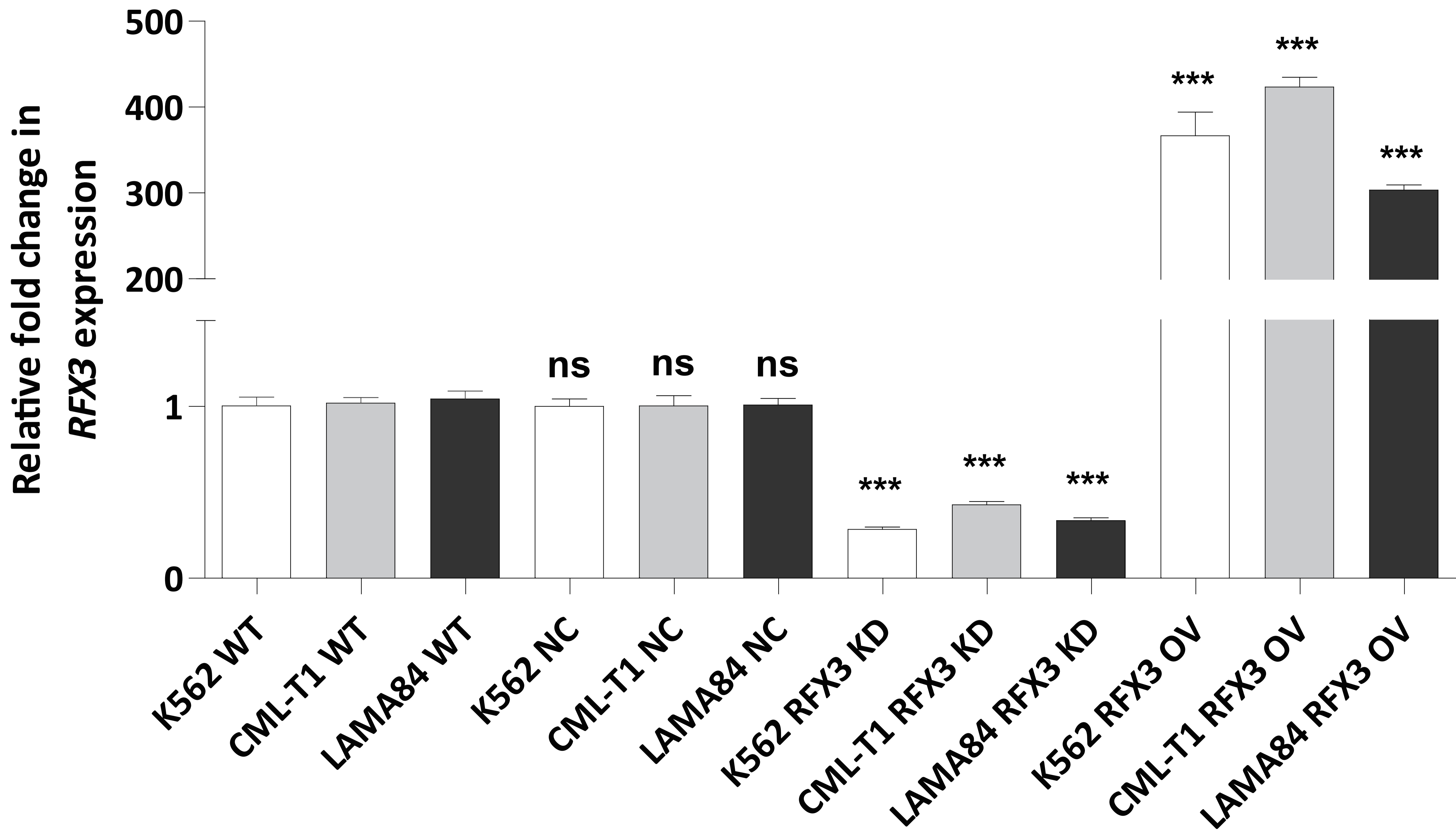

Supplement: S2 Fig — Relative transcript levels of RFX3 were quantified by RT-qPCR and normalized with a house-keeping gene GAPDH. The results show are representative of at least three independent experiments. Statistical analysis was performed using Student’s t-test. ***p < 0.001, ns–not significant. Error bars indicate SD values. (PDF) [file pone.0218968.s002.pdf]
